# Aggregation, Sedimentation and Dissolution of Cu(OH)_2_-Nanorods-Based Nanopesticide in Soil Solutions

**DOI:** 10.3390/nano12213844

**Published:** 2022-10-31

**Authors:** Zhenlan Xu, Qing Tang, Aimei Hong, Lingxiangyu Li

**Affiliations:** 1 Institute of Agro-Product Safety and Nutrition, Zhejiang Academy of Agricultural Sciences, Hangzhou 310021, China; 2 Department of Chemistry, Zhejiang Sci-Tech University, Hangzhou 310018, China; 3 School of Environment, Hangzhou Institute for Advanced Study, University of Chinese Academy of Sciences, Hangzhou 310024, China

**Keywords:** soil solutions, aggregation, sedimentation, Cu(OH)_2_ nanopesticide, dissolution

## Abstract

Along with the development of nanotechnology, nanomaterials have been gradually applied to agriculture in recent years, such as Cu(OH)_2_-nanorods-based nanopesticide, an antibacterial agrochemical with a high efficacy. Nevertheless, knowledge about physical stability of Cu(OH)_2_ nanopesticide in soil solutions is currently scarce, restricting comprehensive understanding of the fate and risk of Cu(OH)_2_ nanopesticide in the soil environment. Herein we investigated aggregation, sedimentation and dissolution of Cu(OH)_2_ nanopesticide in soil solutions extracted from three different soil samples, wherein commercial Cu(OH)_2_ nanopesticide formulation (NPF), as well as its active ingredient (AI) and laboratory-prepared Cu(OH)_2_ nanorods (NR) with similar morphology as AI, were used as model Cu(OH)_2_ nanopesticides. We found that NPF compared to AI showed less extents of aggregation in ultrapure water due to the presence of dispersing agent in NPF. Yet, moderated aggregation and sedimentation were observed for Cu(OH)_2_ nanopesticide irrespective of NPF, AI or NR when soil solutions were used instead of ultrapure water. The sedimentation rate constants of AI and NPF were 0.023 min^−1^ and 0.010 min^−1^ in the ultrapure water, whereas the rate constants of 0.003–0.021 min^−1^ and 0.002–0.007 min^−1^ were observed for AI and NPF in soil solutions, respectively. Besides aggregation and sedimentation, dissolution of Cu(OH)_2_ nanopesticide in soil solutions was highly dependent on soil type, wherein pH and organic matter played important roles in dissolution. Although the final concentrations of dissolved copper (1.08–1.37 mg/L) were comparable among different soil solutions incubating 48 mg/L of AI, NPF or NR for 96 h, a gradual increase followed by an equilibrium was only observed in the soil solution from acidic soil (pH 5.16) with the low content of organic matter (1.20 g/kg). This work would shed light on the fate of Cu(OH)_2_ nanopesticide in the soil environment, which is necessary for risk assessment of the nanomaterials-based agrochemical.

## 1. Introduction

Along with the rapid development of nanotechnology, nanomaterials-based agrochemicals have been designed and prepared by researchers in recent years, showing a great potential for sustainable agriculture in view of their benefits [1,2]. Among nanomaterials-based agrochemicals, nanopesticides are produced and used experimentally in the field to control pest and culture plants efficiently [3]. In general, nanopesticides can be roughly classified into two types [4]: (i) nanosized substances coated with molecular active ingredients such as bifenthrin nanoformulations [5]; and (ii) nanosized active ingredients such as Cu(OH)_2_ nanopesticides [6]. Due to specific physiochemical properties of nanosized substances or active ingredients, nanopesticides relative to conventional pesticides exhibit increased stability, controlled release of active ingredients, superior efficacy, lower dose requirement, and decreasing residues [7]. In this context, Cu(OH)_2_-nanorods-based nanopesticide (denoted as Cu(OH)_2_ nanopesticide) has been available in the market in China, implying that an agricultural application of Cu(OH)_2_ nanopesticide to soil is highly possible.

Once Cu(OH)_2_ nanopesticide has entered soil it is subject to reactions with soil solutions, wherein Cu(OH)_2_ nanorods, the active ingredient of Cu(OH)_2_ nanopesticide, would likely undergo physiochemical transformation. Our recent study showed that Cu(OH)_2_ nanopesticide can affect soil bacterial community and enzyme activity significantly, wherein commercial Cu(OH)_2_ nanopesticide relative to laboratory-prepared Cu(OH)_2_ nanorods exhibited different impacts on the bacterial community [8]. Furthermore, we documented that the laboratory-prepared Cu(OH)_2_ nanorods can transform into CuS nanoparticles in environmental water with dissolved sulfides through in situ formation of CuO nanorods as intermediates, resulting in great changes in the antibacterial activity of Cu(OH)_2_ nanopesticide [9]. Nevertheless, it remains unknown whether Cu(OH)_2_ nanorods can keep physical stability in soil solutions, which is related to the efficacy of Cu(OH)_2_ nanopesticide. Aggregation, sedimentation and dissolution attracted extensive attention in previous studies, demonstrating that physical stability of nanomaterials in the environment is dramatically affected by various processes and environmental factors [10,11,12]. Aggregation, sedimentation and dissolution of CuO nanoparticles in environmental water and soil solutions have been investigated, showing the size-dependent stability [11,13]. However, little is known about the physical stability of Cu(OH)_2_ nanorods in soil solutions even though Cu(OH)_2_ nanopesticide has been experimentally used in the field, restricting an efficacy assessment of Cu(OH)_2_ nanopesticide. In particular, soil solutions are highly dependent on soil properties such as soil pH, organic matter content, and soil texture [14]. Thus, it is important to investigate aggregation, sedimentation and dissolution of Cu(OH)_2_ nanorods in soil solutions extracted from different soils, to evaluate the physical stability of Cu(OH)_2_ nanopesticide comprehensively.

Herein, soil solutions were collected from three soils with different basic properties in this study. Aggregation, sedimentation and dissolution of commercial Cu(OH)_2_ nanopesticide formulation (denoted as NPF) and its active ingredient, namely commercial Cu(OH)_2_ nanorods (denoted as AI), were systematically investigated. In addition, the laboratory-prepared Cu(OH)_2_ nanorods (denoted as NR) with similar morphology to AI were also examined, to further understand the environmental behavior of Cu(OH)_2_ nanorods in soil solutions. This work would improve our knowledge on the stability of Cu(OH)_2_ nanopesticide in the soil environment.

## 2. Materials and Methods

### 2.1. Chemicals

The commercial Cu(OH)_2_ nanopesticide formulation (NPF) and its active ingredient (AI), namely commercial Cu(OH)_2_ nanorods as well as its dispersing agent (denoted as DA), were purchased from Zhejiang Ruili Biotechnology Inc (Zhejiang, China). According to the information of NPF from the supplier, the NPF and AI account for about 77% (weight) and 23% (weight), respectively. We also prepared Cu(OH)_2_ nanorods with similar morphology as the AI in the laboratory. The NR was prepared in the laboratory using the method described in our previous study [9], and the morphology of NPF, AI and NR was shown through transmission electron microscope (TEM) images. The morphology of NFP, AI and NR is shown in the Supplementary Materials (Figure S1). Both NPF and AI were roughly rod-like shaped with 80–140 nm in diameter and 160–350 nm or more in length, and NR exhibited nanorod-shaped morphology with 10–16 nm in diameter and 125–380 nm in length. Characterization such as X-ray diffraction (XRD) and zeta potential was conducted in our recent studies which showed the details [6,8]. The specific surface areas based on the Brunauer, Emmett and Teller (BET) theory were 44.2 m^2^/g, 71.6 m^2^/g and 92.6 m^2^/g for NPF, AI, and NR, respectively. Copper sulfate, sodium hydroxide and ethylenediaminetetraacetic acid disodium salt were purchased from Aladdin (Shanghai, China).

### 2.2. Soil Sampling and Soil Solution Extraction

Three soil samples (surface layer, 0–20 cm) were manually collected from the campus of the Zhejiang Sci-Tech University located at Hangzhou city (China), an unnamed hill located at Anji city (China), and farmland located at Tongren city (China), respectively. Soil samples were air dried, ground and passed through a 1 mm sieve to remove plant roots, stones and plastics. The basic physiochemical properties of soil samples are shown in Table S1 (Supplementary Materials). The pH values of soil were 8.35, 5.16 and 6.83, corresponding to organic matter contents of 29.8, 1.20 and 236.2 g/kg, respectively.

Soil solutions were collected through the method developed by Klitzke et al. with minor modifications [15]. In brief, 20 g soil sample was added to 200 mL of ultrapure water (18.2 MΩ cm^−1^) in a glass vessel that was fixed on an end-over-end shaker (60 rpm) for 16 h, followed by centrifugation at 6000 rpm for 15 min. The supernatant was filtered through a 0.45 μm polyether sulphone filter, followed by storage at 4 °C until usage. The background concentration of Cu in the soil solutions was determined using inductively coupled plasma optical emission spectrometry (ICP-OES, Thermo Scientific, Waltham, MA, USA) after the solution was acidified using the concentrated HNO_3_.

### 2.3. Aggregation and Sedimentation of Cu(OH)_2_ Nanopesticide in Soil Solutions

A certain amount of NPF, AI or NR was added into soil solutions, yielding stock solutions with the concentration of copper at 240 mg/L, followed by sonication (KQ-600E, 40 kHz, 600 W) at 0 °C (ice-water mixture) for 15 min to disperse the stock solution completely. Then 2 mL of stock solution was mixed with 8 mL of soil solution, wherein the final concentration of copper was 48 mg/L. It should be noted that the concentration of copper used at 48 mg/L was proposed through calculation based on actual application of Cu(OH)_2_ nanopesticide at up to 11.8 kg ha^−1^, wherein a depth penetration of 1 cm soil results in concentrations of copper about 50 mg/kg [16]. The aggregation of NPF, AI or NR was investigated through dynamic light scattering (DLS), wherein a Nano ZS-90 zetasizer (Malvern, UK) was used to record data every 2 min for 1.5 h. The sedimentation of NPF, AI or NR was monitored through UV-vis spectrophotometer (UV-2700, Shimadzu, Japan) every 2 min for 1.5 h. The absorbance intensity gradually decreased with an increase in incubation time, which is attributed to sedimentation of NPF, AI or NR. Thus, the change in the absorbance intensity as a function of incubation time can be correlated with the normalized concentration of NPF, AI or NR in soil solutions [11]. A semi-empirical model was developed to fit the sedimentation kinetics [17], which can optimize the sedimentation rate and the proportion of suspended NPF, AI or NR. As shown in Equation (1), C represents the copper concentration (mg/L) at time t (min), and *C*_0_ is the initial concentration (48 mg/L) at time of 0 min. Moreover, *C*_res_ (mg/L) is the concentration of suspended NPF, AI or NR at infinite time, k_sed_ (min^−1^) is the sedimentation rate, and t (min) is the sedimentation time.

(1) $$\frac{C}{C_{0}}=\left(1-\frac{C_{\mathrm{res}}}{C_{0}}\right)e^{-k_{\mathrm{sed}}*t}+\frac{C_{\mathrm{res}}}{C_{0}}$$

### 2.4. Dissolution of Cu(OH)_2_ Nanopesticide in Soil Solutions

Considering the possible dissolution of NPF, AI or NR during aggregation and sedimentation, dissolution as a function of incubation time was evaluated. In brief, 2 mL of soil solution with NPF, AI or NR at an initial copper concentration of 48 mg/L was collected at a certain time (e.g., 0, 15, 30, 60 and 90 min), followed by centrifugal filtration (Amicon Ultra-4, 3 kD, Millipore, Burlington, MA, USA) at 9500 rpm for 15 min, to collect the filtrate. The concentration of dissolved copper in the filtrate was determined through ICP-OES after the solution was acidified using the concentrated HNO_3_. In order to validate the centrifugal filtration at 9500 rpm for 15 min, a recovery efficiency of 48 mg/L ionic copper (e.g., CuSO_4_) solution was 98.6 ± 1.3%, implying that there was a negligible loss of the ionic copper during the centrifugal filtration.

## 3. Results and discussion

### 3.1. Aggregation of Cu(OH)_2_ Nanopesticide in Soil Solutions

**Figure 1:**
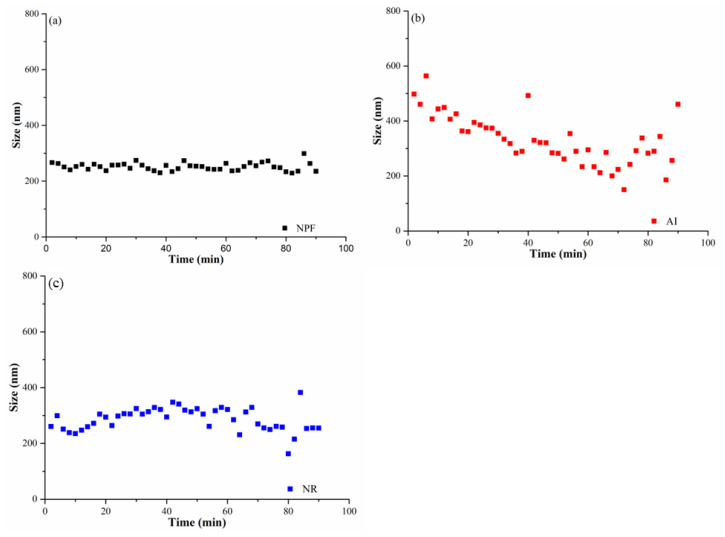
The aggregation of NPF, AI and NR in ultrapure water as a function of time: (**a**) NPF: (**b**) AI: (**c**) NR.

Considering the great differences in the physiochemical property between ultrapure water and soil solution, the aggregation of NPF, AI and NR in ultrapure water was examined first, which can provide idealized information about the stability of Cu(OH)_2_ nanopesticide. As shown in Figure 1a, the hydrodynamic sizes of NPF in the ultrapure water keep stable (240–270 nm) throughout the whole incubation time, indicating negligible homoaggregation of NPF. Nevertheless, obvious aggregation behavior of AI was observed in the ultrapure water, wherein the largest hydrodynamic sizes (about 500 nm) of AI were observed at the first stage of incubation time (e.g., 0–6 min) (Figure 1b). Along with an increase in the incubation time, the hydrodynamic size then gradually decreased (Figure 1b), which might be attributed to the fact that some aggregated particles would likely settle down in the ultrapure water. Nevertheless, the hydrodynamic size increased from 170 nm to 465 nm after an incubation of 70 min (Figure 1b), indicating that homoaggregation of AI continuously occurred. Given the fact that NPF is composed of AI and DA, we proposed that DA would be in favor of stabilizing AI in the ultrapure water, which is an important role of DA for the NPF. As shown in Figure 1c, the hydrodynamic sizes are around 250 nm throughout the whole incubation time, being comparable to those of NPF (Figure 1a). In total, it is obvious that NPF is more stable than AI in the ultrapure water.

Aggregation of NPF in the AJ soil solution was observed (Figure S2a in the Supplementary Materials), wherein the hydrodynamic size of NPF increased from 310 nm to 1270 nm within the initial 36 min, followed by a gradual decrease in hydrodynamic size (Figure S2a), which might be attributed to sedimentation of the aggregated particles. Despite this, the hydrodynamic size of NPF in the HZ soil solution was stable (Figure S3a), being different from the phenomenon of NPF in the AJ soil solution, suggesting that the soil property would dramatically affect the aggregation of NPF in soil solutions. The superior stability of NPF in the HZ soil solution might be attributed to the role of natural organic matter, since high content of organic matter was determined in HZ soil compared to AJ soil (Table S1). Accordingly, the content of natural organic matter in the HZ soil solution (12.6 mg/L) was higher than that of AJ soil solution (1.8 mg/L). Previous studies have demonstrated the great role of natural organic matter in stabilizing nanomaterials in aqueous solutions [18,19]. Similarly, aggregation of AI or NR in the AJ soil solution was more serious than that in HZ soil solution (Figures S2b,c and S3b,c), which is also reasonable due to higher content of natural organic matter in the latter. Moreover, aggregation of NR prepared in the laboratory was a little slighter than that of AI irrespective of AJ or HZ soil solution, which is consistent with the phenomenon of aggregation occurring in ultrapure water.

### 3.2. Sedimentation of Cu(OH)_2_ Nanopesticide in Soil Solutions

**Figure 2:**
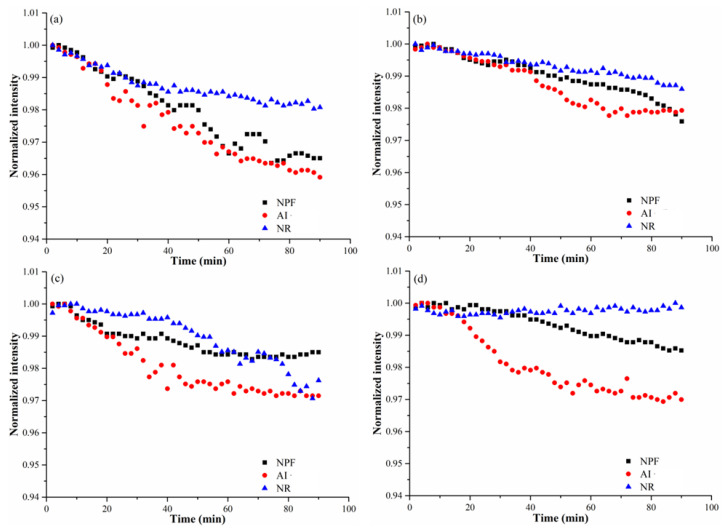
The sedimentation of NPF, AI and NR in different solutions as a function of time: (**a**) Ultrapure water; (**b**) HZ soil solution; (**c**) AJ soil solution; (**d**) TR soil solution.

Due to aggregation of Cu(OH)_2_ nanopesticide in soil solutions, the sedimentation would likely occur. As shown in Figure 2, great differences in the sedimentation behavior of NPF, AI and NR are observed irrespective of in ultrapure water or in soil solutions. About 96.5% of NPF and 95.5% of AI were found suspended in the ultrapure water after settling for 90 min, while about 98% of NR can still suspend in the ultrapure water (Figure 2a). This implies good stability of NR in the ultrapure water, being well consistent with the aggregation behavior, wherein negligible aggregation was observed for NR. In addition, AI showed serious sedimentation behavior in ultrapure water, which can explain the decrease in the hydrodynamic sizes of AI (Figure 1b). Accordingly, the constants of sedimentation rate were 0.010, 0.023 and 0.03 min^−1^ for NPF, AI and NR, respectively (Table 1).

Once NPF, AI or NR was suspended in soil solutions instead of ultrapure water, slower sedimentation behavior was observed (Figure 2b–d), indicating that larger proportions of Cu(OH)_2_ nanopesticide were suspended in soil solutions. In HZ soil solution, for example, about 97.5% of NPF and 97.8% of AI were found suspended after settling for 90 min, wherein NR exhibited a large proportion (e.g., 98.7%) (Figure 2b). The increase in the proportion of suspended AI was 2.3% when the HZ soil solution was used instead of ultrapure water, resulting in the constancy of the sedimentation rate ranging from 0.023 min^−1^ to 0.003 min^−1^. This might be due to the role of natural organic matter in the soil solution since the natural organic matter was documented to be able to moderate sedimentation of nanomaterials through modifying the surface of nanomaterials in the aqueous environment [20]. For example, when concentrations of natural organic matter ranged from 0.4 mg/L to 37 mg/L, the proportion of suspended CeO_2_ nanoparticles in water increased from 36% to 87% after 12 d of settling [21]. Meanwhile, soil pH may also play a role in affecting the sedimentation behavior, especially considering the fact that only AJ soil showed acidic value (pH 5.16) which can accelerate dissolution of Cu(OH)_2_ and make the Cu(OH)_2_ nanopesticide unstably. Previous studies have already documented that metal-based nanomaterials displayed significantly higher solubility at low pH than at neutral pH [22,23].

### 3.3. Dissolution of Cu(OH)_2_ Nanopesticide in Soil Solutions

**Figure 3:**
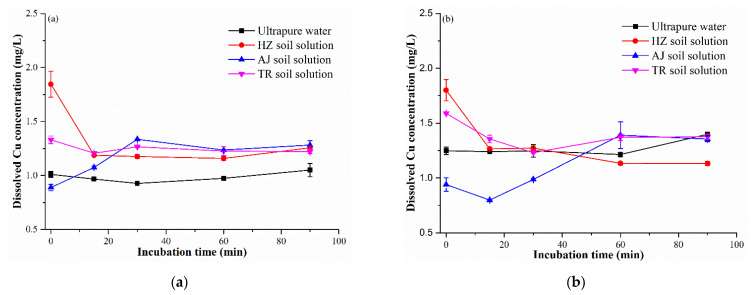
Dissolved Cu concentration as a function of incubation time: (**a**) NPF; (**b**) AI.

Along with the aggregation and sedimentation, dissolution of Cu(OH)_2_ nanopesticide was also examined, especially the possible differences in the dissolution behavior between NPF and AI, which would show the effects of dispersing agent on the dissolution of AI. As shown in Figure 3, the concentration of dissolved Cu in the ultrapure water is stable throughout the whole incubation time irrespective of NPF or AI. The dissolved Cu may be residual during the preparation of NPF and AI, which can be released immediately when NPF and AI were added into ultrapure water, which gives the reason for the measurable level of dissolved Cu at 0 min. Although the final concentrations of dissolved copper (1.08–1.37 mg/L) were comparable among different soil solutions incubating 48 mg/L of AI, NPF or NR for 96 h, soil property can affect the dissolution behavior of Cu(OH)_2_ nanopesticide (Figure 3); the concentration of dissolved Cu in the AJ soil solution with NFP or AI gradually increased, followed by an equilibrium, whereas the HZ soil solution with NPF or AI exhibited a decreasing trend, reaching an equilibrium afterward. This might be attributed to the pH values of soil solution between AJ and HZ, wherein the former was an acidic solution, promoting dissolution of Cu(OH)_2_ nanopesticide. Solution pH was documented as an important factor affecting dissolution of metal-based nanomaterials [24]. Negligible differences in the dissolution behavior were observed for NPF and AI (Figure 3), indicating that the dispersing agent in NPF would not affect the dissolution of AI.

## 4. Conclusion

In this study, aggregation and sedimentation of Cu(OH)_2_ nanopesticide such as NPF, AI and NR were investigated in ultrapure water and soil solutions, documenting that aggregation and sedimentation behaviors occurring in ultrapure water were more serious than those in soil solutions, which might be attributed to the occurrence of natural organic matter in soil solutions. Moreover, NPF relative to AI exhibited good stability, irrespective of whether in ultrapure water or in soil solutions, due to the presence of dispersing agent in the NPF. Besides aggregation and sedimentation, dissolution of Cu(OH)_2_ nanopesticide was affected by the basic property of soil solution, such as pH. Taken together, our work can provide valuable information about environmental behavior of Cu(OH)_2_ nanopesticide in soil solutions once it was applied to soil, which would improve the understanding of environmental risk of Cu(OH)_2_ nanopesticide.

## Figures and Tables

**Table 1:** The sedimentation parameters of NPF, AI, or NR in different solutions.

Solution	Samples	k_sed_ (min^−1^)	*C*_res_/*C*_0_	R^2^
Ultrapure water	NPF	0.010	0.826	0.954
	AI	0.023	0.929	0.973
	NR	0.003	0.978	0.976
HZ soil solution	NPF	0.002	0.986	0.786
	AI	0.003	0.859	0.932
	NR	0.002	0.913	0.899
AJ soil solution	NPF	0.005	0.981	0.948
	AI	0.021	0.965	0.945
	NR	- ^a^	- ^a^	- ^a^
TR soil solution	NPF	0.007	0.971	0.910
	AI	0.016	0.959	0.936
	NR	- ^a^	- ^a^	- ^a^

^a^ represents that the equation is not suitable for the data, showing negative value for R^2^.

## Supplementary Materials

The following supporting information can be downloaded at: https://www.mdpi.com/article/10.3390/nano12213844/s1, Figure S1: TEM images of NPF (a), AI (b) and NR (c) used in this study; Figure S2: The aggregation of NPF, AI and NR in the AJ soil solution as a function of time. (a) NPF. (b) AI. (c) NR; Figure S3: The aggregation of NPF, AI and NR in the HZ soil solution as a function of time. (a) NPF. (b) AI. (c) NR. Table S1: Basic physiochemical properties of soil used in this study.
