# Supplementary material for: Aggregation, Sedimentation and Dissolution of Cu(OH)2-Nanorods-Based Nanopesticide in Soil Solutions
Source: Nanomaterials (Basel). 2022 Oct 31;12(21):3844. doi: 10.3390/nano12213844 (PMC9657550; doi:10.3390/nano12213844)
Supplement: Supplementary file 1 [file nanomaterials-12-03844-s001.zip › nanomaterials-1987145-supplementary.pdf]

Supplementary materials

Aggregation, Sedimentation and Dissolution of $Cu(OH)_2$-Nanorods-Based Nanopesticide in Soil Solutions

Zhenlan Xu [1], Qing Tang [2], Aimei Hong [2] and Lingxiangyu Li [3,*]

[1] Institute of Agro-Product Safety and Nutrition, Zhejiang Academy of Agricultural Sciences, Hangzhou 310021, China
[2] Department of Chemistry, Zhejiang Sci-Tech University, Hangzhou 310018, China
[3] School of Environment, Hangzhou Institute for Advanced Study, University of Chinese Academy of Sciences, Hangzhou 310024, China
* Correspondence: lingxiangyu.li@ucas.ac.cn

Table S1. Basic physiochemical properties of soil used in this study

Location	pH	Total N (g/kg)	Organic matter (g/kg)	Dissolved N (mg/kg)	Bioavailable P (mg/kg)	EC (uS/cm)	Dissolved salt (g/kg)	Total S (g/kg)	CEC (cmol/kg)
HZ	8.35	0.470	29.8	32.3	33.9	239	0.65	4.79	8.11
AJ	5.16	0.010	1.20	3.20	3.66	6.33	0.00	9.30	3.52
TR	6.83	8.595	236.2	336.8	25.5	342	0.93	21.1	30.5

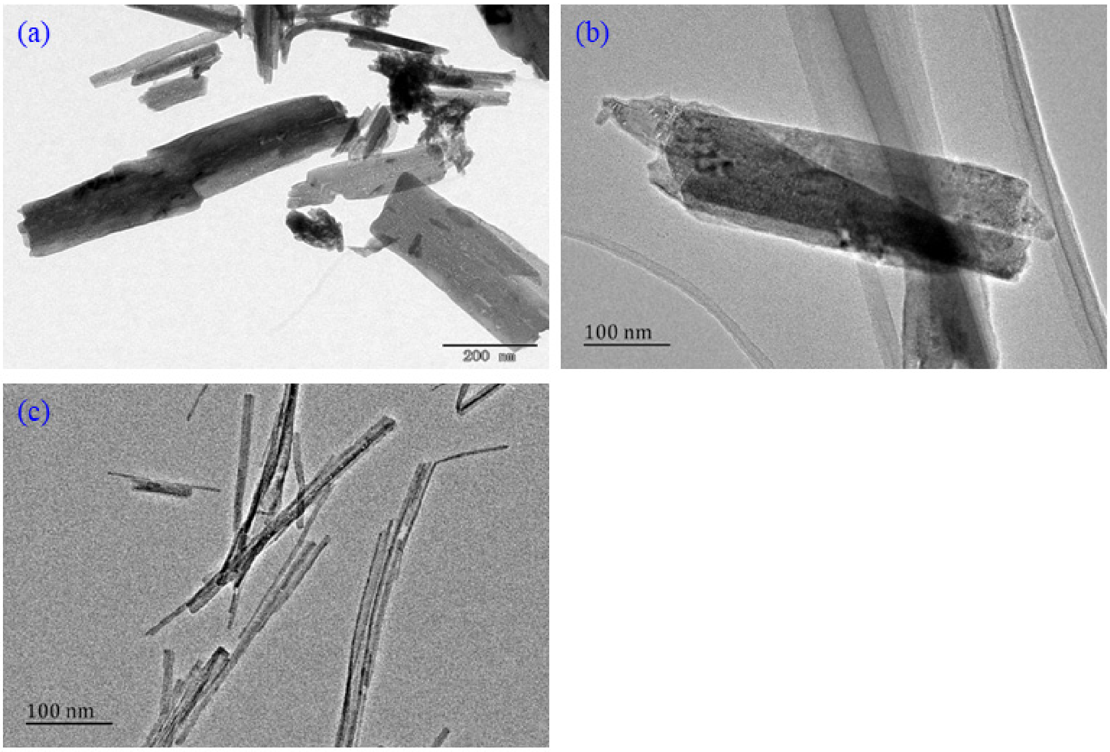

Figure S1. TEM images of NPF (a), AI (b) and NR (c) used in this study.

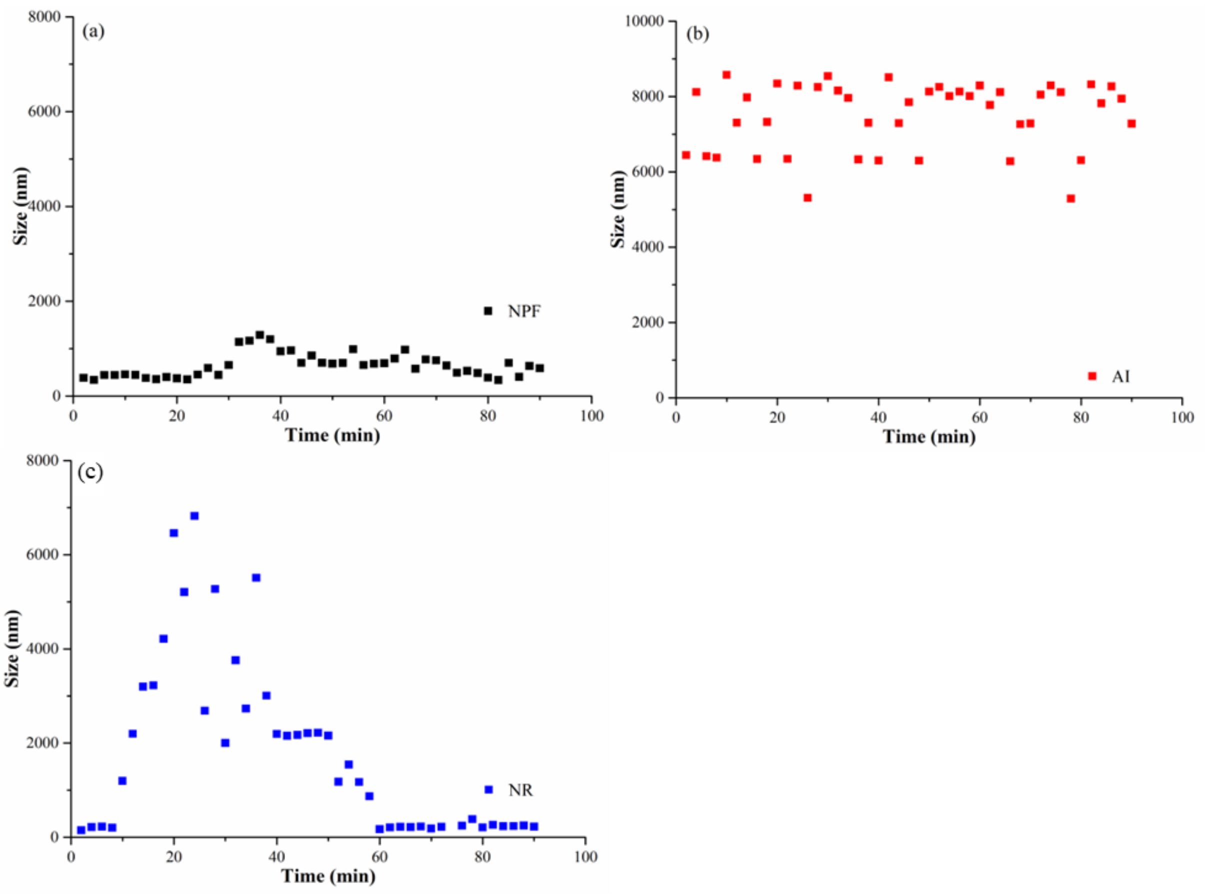

Figure S2. The aggregation of NPF, AI and NR in the AJ soil solution as a function of time. (a) NPF. (b) AI. (c) NR.

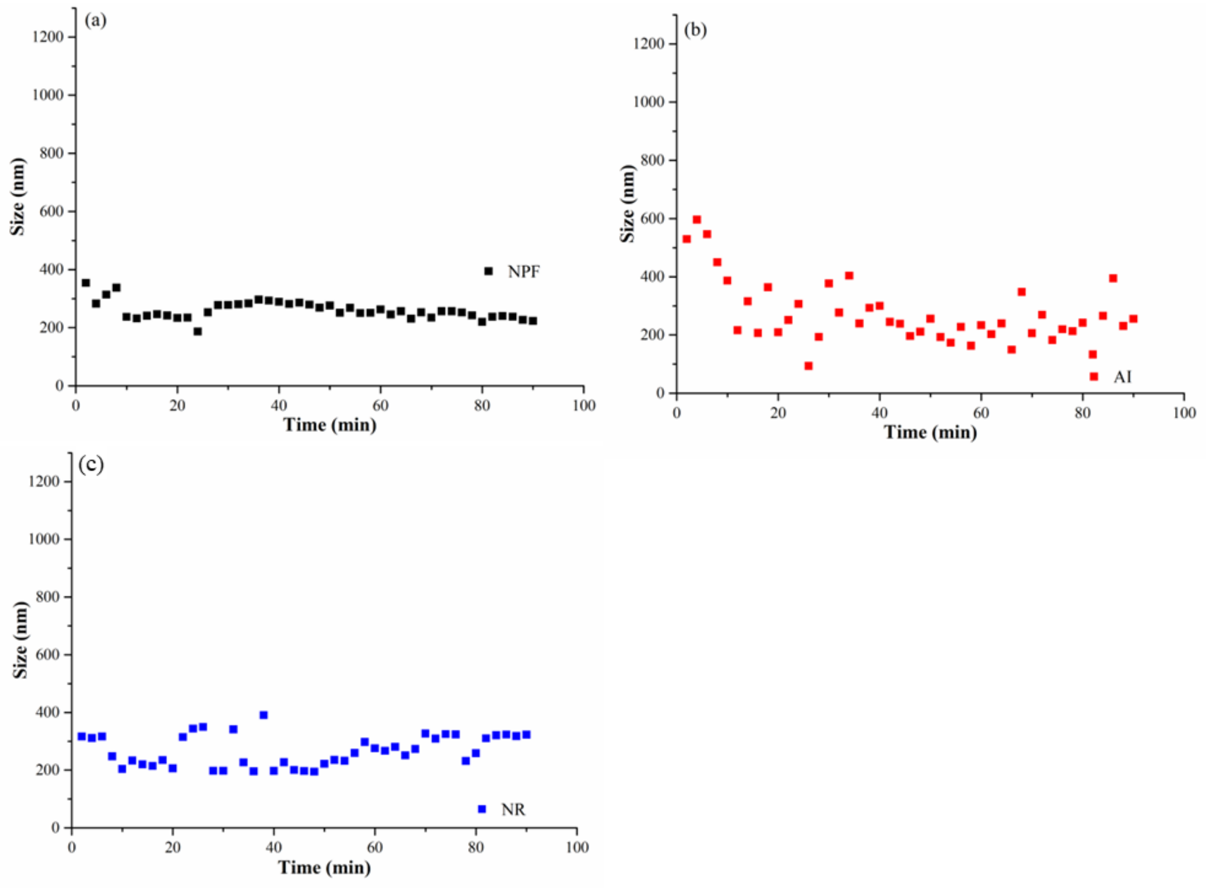

Figure S3. The aggregation of NPF, AI and NR in the HZ soil solution as a function of time. (a) NPF. (b) AI. (c) NR.
